# Risk factors for recurrent infection in the surgical treatment of infected massive endoprostheses implanted for musculoskeletal tumours

**DOI:** 10.1186/s13018-022-03446-1

**Published:** 2023-01-30

**Authors:** Tariq Azamgarhi, Simon Warren, Will Aston, Rob Pollock, Craig Gerrand

**Affiliations:** 1 Pharmacy Department, Royal National Orthopaedic Hospital NHS Trust, Brockley Hill, Stanmore, HA7 4LP UK; 2 Bone Infection Unit, Royal National Orthopaedic Hospital NHS Trust, Brockley Hill, Stanmore, HA7 4LP UK; 3 Division of Orthopaedic Oncology, Royal National Orthopaedic Hospital NHS Trust, Brockley Hill, Stanmore, HA7 4LP UK; 4 The Royal Free Hospital NHS Foundation Trust, Hampstead, London, UK

## Abstract

**Background:**

Infection is a devastating complication of endoprosthetic replacement (EPR) in orthopaedic oncology. Surgical treatments include debridement and/or one- or two-stage exchange. This study aims to determine the infection-free survival after surgical treatment for first and recurrent EPR infections and identify the risk factors associated with infection recurrence.

**Methods:**

This single-centre cohort study included all patients with primary bone sarcomas or metastatic bone disease treated for infected EPR between 2010 and 2020. Variables included soft tissue status using McPherson classification, tumour type, silver coating, chemotherapy, previous surgery and microorganisms identified. Data for all previous infections were collected.

Survival analysis, with time to recurrent infection following surgical treatment, was calculated at 1, 2 and 4 years. Cox regression analysis was used to assess the influence of different variables on recurrent infection.

**Results:**

The cohort included 99 patients with a median age of 44 years (29–58 IQR) at the time of surgical treatment. The most common diagnoses were osteosarcoma and chondrosarcoma.

One hundred and thirty-three surgical treatments for first or subsequent infections were performed. At 2 years of follow-up, overall success rates were as follows: two-stage exchange 55.3%, one-stage exchange 45.5%, DAIR with an exchange of modular components 44.6% and DAIR without exchange of modular components 24.7%.

Fifty-one (52%) patients were infection-free at the most recent follow-up. Of the remaining 48 patients, 27 (27%) were on antibiotic suppression and 21 (21%) had undergone amputation.

Significant risk factors for recurrent infection were the type of surgical treatment, with debridement alone as the highest risk (HR 4.75: 95%CI 2.43–9.30; *P* < 0.001); significantly compromised soft tissue status (HR 4.41: 95%CI 2.18–8.92; *P* = 0.001); and infections due to *Enterococcus spp.*. (HR 7.31: 95%CI 2.73–19.52); *P* = 0.01).

**Conclusions:**

Two-stage exchange with complete removal of all components where feasible is associated with the lowest risk of recurrent infection. Poor soft tissues and enterococcal infections are associated with higher risks of recurrent infection. Treatment demands an appropriate multidisciplinary approach. Patients should be counselled appropriately about the risk of recurrent infection before embarking on complex treatment.

## Introduction

Limb salvage with endoprosthetic replacement (EPR) achieves good functional outcomes in the surgical treatment of musculoskeletal tumours. Infection following EPR is devastating for patients leading to severe pain, poor function, reduced quality of life, and even death [1–3]. Definitive treatment of infection is based on eradication of the biofilm through debridement and implant exchange. This is challenging surgery associated with further complications. Even with implant exchange, failure rates are unacceptably high, varying from 20 to 50% [4, 5]. The risk factors for recurrent infection remain poorly defined, although immunocompromise in oncology patients, poor soft tissue cover and the large size of EPRs may contribute [4–6].

This study assesses the success of surgical treatment of infected EPRs, the microbiological profile of infection and identifies the risk factors associated with recurrent infection. Our aim is to provide clinicians with data to inform decisions about the surgical treatment of infected EPRs based on the experience at our high-volume centre.

## Methods and study population

Patients of all ages treated for infected EPRs at our centre between 2010 and 2020 were included in this study. Patients undergoing primary implantation of an EPR for primary bone tumours (PBT) or metastatic bone disease (MBD) were identified from electronic hospital records and clinical coding.

First and recurrent EPR infections were included, and those managed with amputation alone or antibiotic suppression at the first episode of infection were excluded. Electronic patient records were reviewed for information on all previous surgical treatments for infected EPR dating back to the index EPR procedure. For each surgical treatment, the following clinical and microbiological data were collected: details of tumour type and initial prosthesis implanted, history of radiotherapy and chemotherapy, presentation of infection, soft tissue status, infecting organism, surgical treatment and follow-up. The condition of the local soft tissues at the time of surgery was categorised using the staging system proposed by McPherson, which considers the following compromising factors: active infection for longer than 3 months; previous radiotherapy; soft tissue loss; the presence of a fistula or subcutaneous abscess and prior periarticular fracture [7]. The numbers of compromising factors were used to categorise local soft tissue status as: ‘uncompromised’ if none were present; ‘compromised’ if one or two were present; and ‘significantly compromised’ if three or more were present.

### Surgery

The consultant surgeon decided on the choice of surgical treatment often in consultation with colleagues. Patients who had an initial washout to control sepsis before going on to have a definitive surgical treatment for infection were classified under the definitive procedure. The approach to surgery in our centre is to perform a comprehensive debridement and excision of infected and nonviable bone or soft tissues with tissue sampling for microbiological analysis plus or minus implant removal/exchange. Surgical interventions were classified as one-stage; two-stage; debridement, antibiotics, and implant retention (DAIR) with exchange of modular components; DAIR without exchange of modular components; and excision arthroplasty. One-stage procedures involved the exchange of all prosthetic components, whereas two-stage procedures involved the implantation of a spacer and prolonged systemic antibiotic therapy followed by a second-stage implantation of a new prosthesis. Patients intended for a two-stage exchange who had the first-stage but did not undergo a second-stage procedure were categorised as two-stage exchange (without reimplantation). We defined DAIR as debridement and change of modular components with retention of at least one prosthetic stem. DAIR without exchange of modular components did not include a change of any modular components or prosthetic stems but did involve debridement of soft tissues and prolonged systemic antimicrobial therapy. Excision arthroplasty involved the removal of the implant without further reconstruction.

The Stanmore METS® (Modular Endoprosthetic Tumour System) (Stanmore Implants Worldwide, Stryker Corporation, Elstree, UK) was the most frequently used prosthesis. Some implants incorporated an Agluna® antimicrobial ionic silver surface coating.

All patients received 6 to 12 weeks of pathogen-directed systemic antimicrobial treatment based on microbiological culture and sensitivities. Typically, the first 6 weeks were intravenous, with or without an oral adjunct, followed by a switch to oral antibiotics for an intended 12 weeks. Staphylococcal and culture-negative infections were usually treated with teicoplanin for 6 weeks, with the addition of oral rifampicin, followed by an oral switch, most commonly either to doxycycline or ciprofloxacin in combination with rifampicin for 12 weeks. After May 2017, our microbiologists considered exclusively oral antibiotic treatment based on the findings of the OVIVA Trial [8].

### Definition of recurrent infection

We defined recurrent infection as follows: 1. infection with positive cultures of the same or different organisms from periprosthetic samples or an aspirate; 2. wound or sinus drainage recurring or persisting for 3 months beyond the index procedure for infection; or 3. a requirement for further surgery for an infection.

### Statistical analysis

Descriptive statistics were used to report continuous and categorical variables. Kaplan–Meier survivorship curves with recurrent infection as an endpoint were generated for 1-year, 2-year and 4-year follow-up with 95% confidence intervals (CI). The relationship between variables was investigated using a two-stage process. Firstly, a univariate Cox analysis was performed to analyse the relationship between each factor and recurrent infection. Significant variables (if *P* < 0.1) were then included in a multivariate Cox regression analysis. A backwards selection procedure was performed to retain factors associated with the outcome in the final model. Non-significant factors were omitted one at a time until only factors showing an association with the outcome remained. The results of the regression analysis are expressed as hazard ratios with 95% confidence intervals. *P values* ≤ 0.05 were considered statistically significant. Statistical analysis was performed using SPSS version 25.0 (IBM, Chicago, ILs).

## Results

### Characteristics of the cohort

Ninety-nine patients were treated for infected EPR between 2010 and 2020. No patients were lost to follow-up. The median age at surgical treatment (IQR) was 44 years (29–58 IQR). Tumour types were osteosarcoma (47), chondrosarcoma (23), Ewing’s sarcoma (7), other soft tissue/bone sarcoma (7), giant cell tumour of bone (7) and metastatic bone disease (8). The median time to the first infection was 6.1 years (IQR 1.6 – 28.5).

### Surgical treatment

**Fig. 1:**
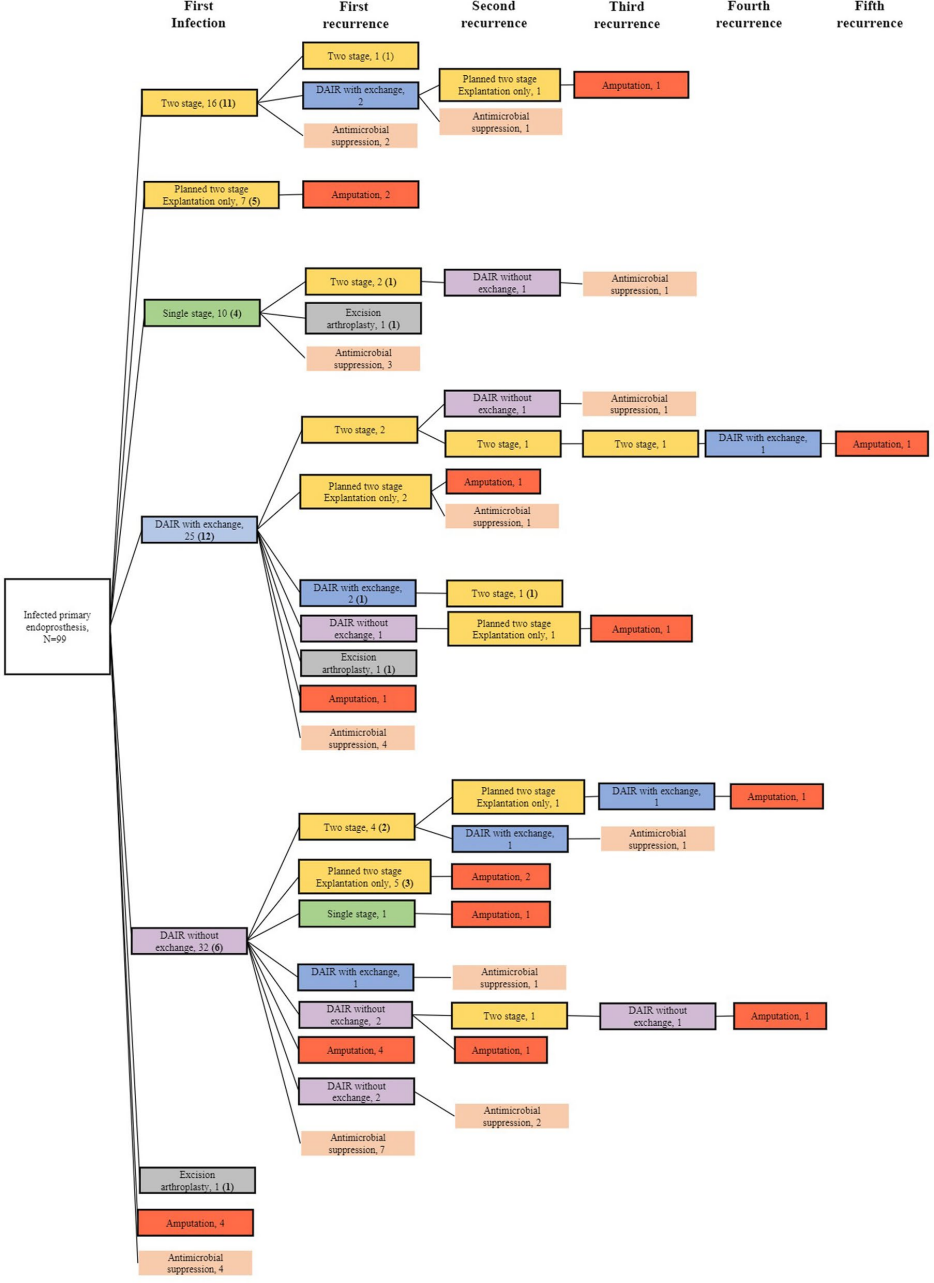
Flow chart showing treatment performed in 99 patients diagnosed with infected primary endoprostheses. The number of cases without infection recurrence after surgical treatment are shown in parentheses

Of 99 first infections, 4 proceeded with antibiotic suppression, 4 underwent amputation, and 91 received surgical treatment (Fig. 1). Patients treated with surgery were followed up for a median of 61 months (IQR 40–86). The success rate of a two-stage exchange reduced at each episode of infection. At the first episode of infection, success rates were 11 of 16 (69%) for two-stage exchange and 5 of 7 (71%) for an incomplete two-stage exchange. However, when treating a second episode of infection, success rates were lower at 4 of 9 (44%) for a completed two-stage exchange and 3 of 7 (43%) for incomplete two-stage exchange.

**Fig. 2:**
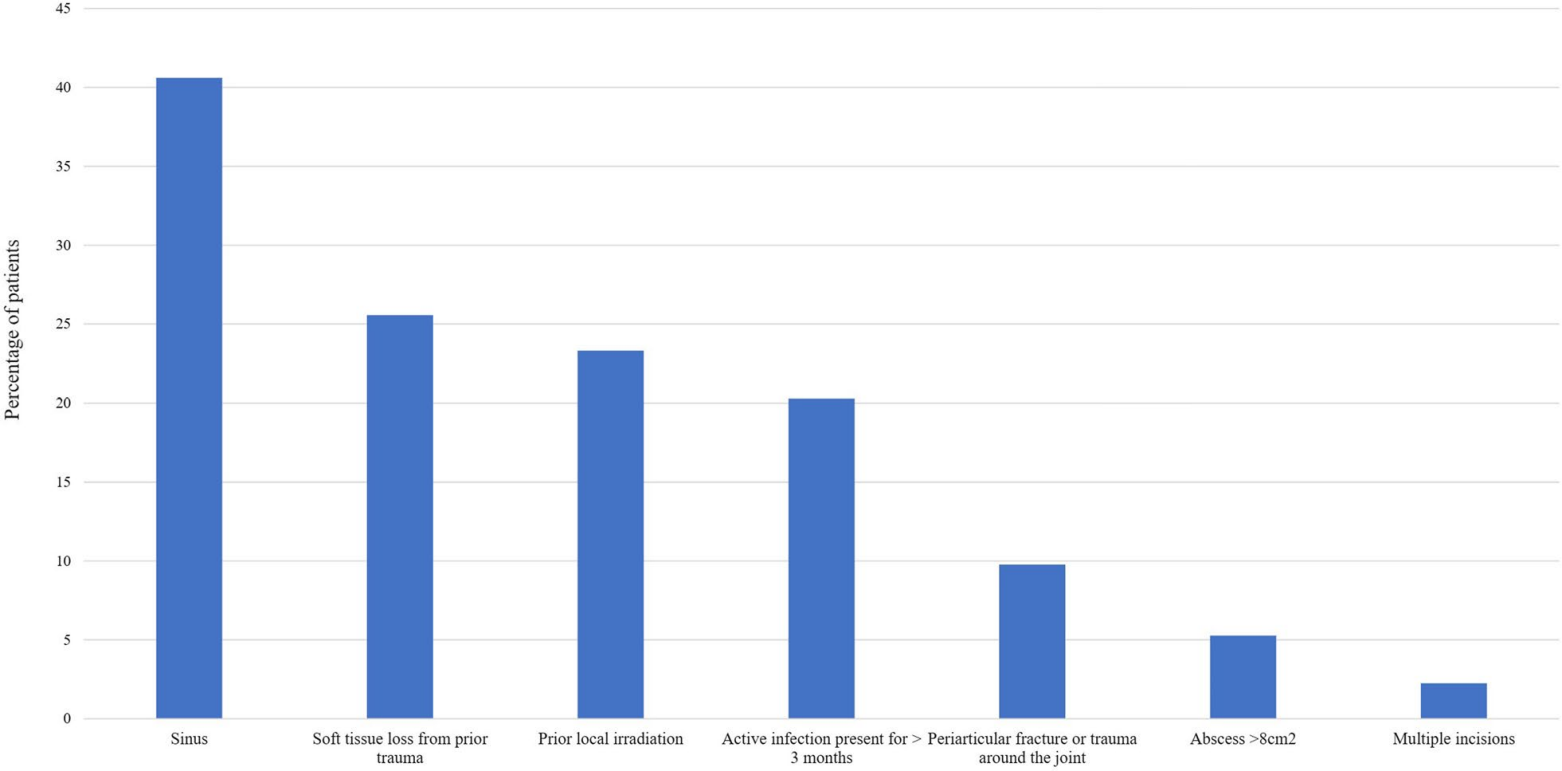
Compromising factors of local soft tissue status classified according to McPherson criteria

Fifty-one (52%) patients were infection-free at the final follow-up. Of the remaining 48 patients, 27 (27%) were on antibiotic suppression, and 21 (21%) had undergone amputation. Compromising soft tissue factors identified at the time of surgery are shown in Fig. 2. Flap coverage by a plastic surgeon was required for 17 surgical treatments (13.3%).

### Microbiology

**Fig. 3:**
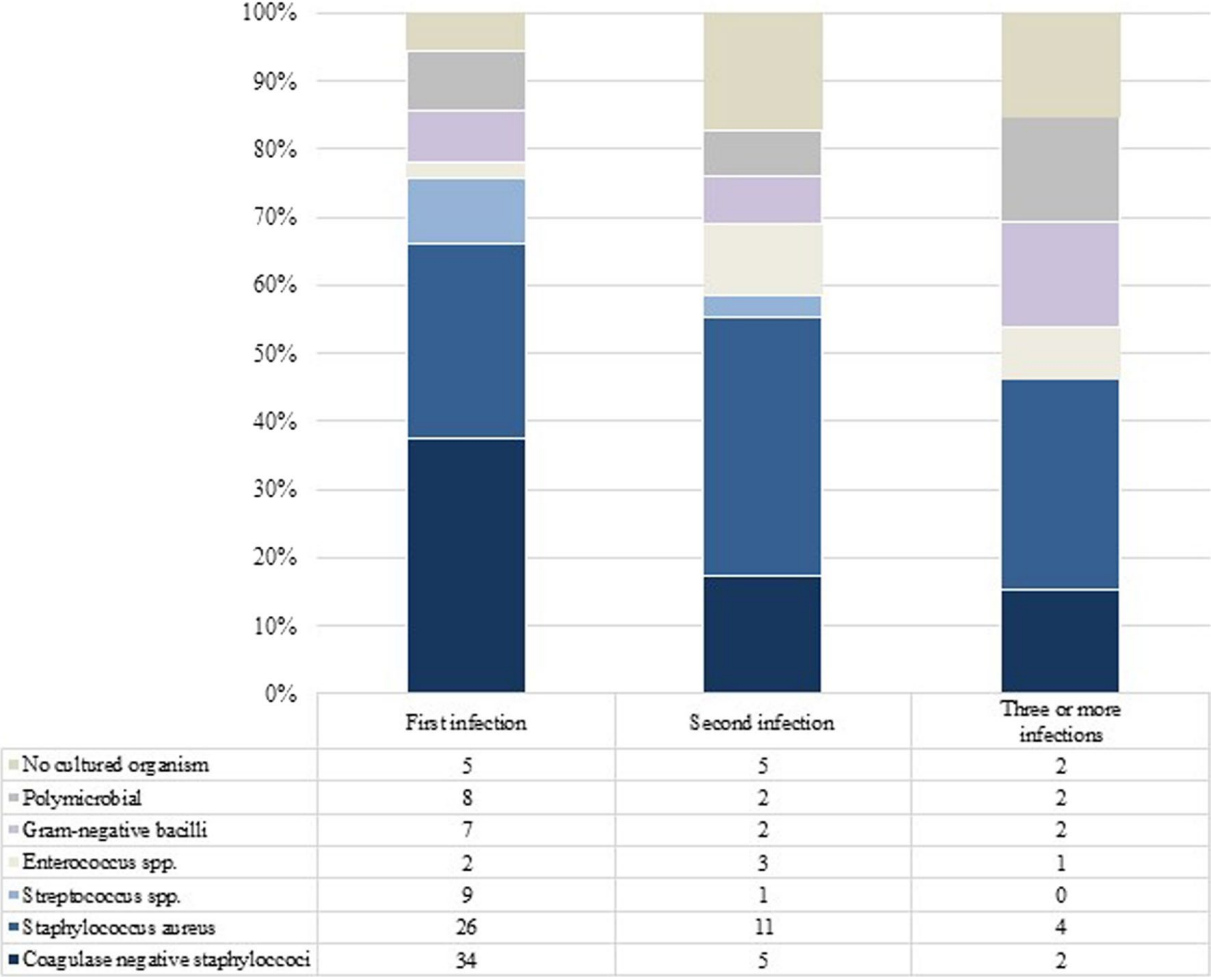
The trend in microorganisms identified at sequential episodes of EPR infection

Organisms were cultured in 91.0% (121 of 133) of infections (Fig. 3).

At first infection, monomicrobial infections were most commonly due to coagulase-negative staphylococci (39.5%, 34 of 86), followed by *Staphylococcus aureus* (30.2%, 26 of 86), *Streptococcus spp.* (10.5%, 9 of 86), Gram-negative bacilli (8.1%, 7 of 86) and *Enterococcus spp*. (2.3%, 2 of 86). 9.3%, 8 of 86) were polymicrobial infections.

The infecting organisms changed with an increasing number of recurrent infections. At the second episode of infection, there was a lower proportion of monomicrobial infections caused by coagulase-negative staphylococci, 20.8% (5 of 24) and one streptococcal infection. In comparison, there were proportionately more monomicrobial infections caused by *Staphylococcus aureus,* 45.8% (11 of 24) and *Enterococcus spp*. 12.5% (3 of 24). After three or more episodes of infection, most were monomicrobial infections caused by *Staphylococcus aureus* (36.4%, 4 of 11), Coagulase-negative staphylococci (18.1%, 2 of 11), Gram-negative bacilli (18.1%, 2 of 11) or *Enterococcus spp*. (9.1%, 1 of 11). 18.1% (2 of 11) were polymicrobial.

57.1% (24 of 42) of recurrent infections were due to the same pathogen group, whereas 26.2% (11 of 42) were due to a different organism group. The remaining 16.7% (7 of 42) of recurrent infections had no cultured organisms.

### Outcome

**Fig. 4:**
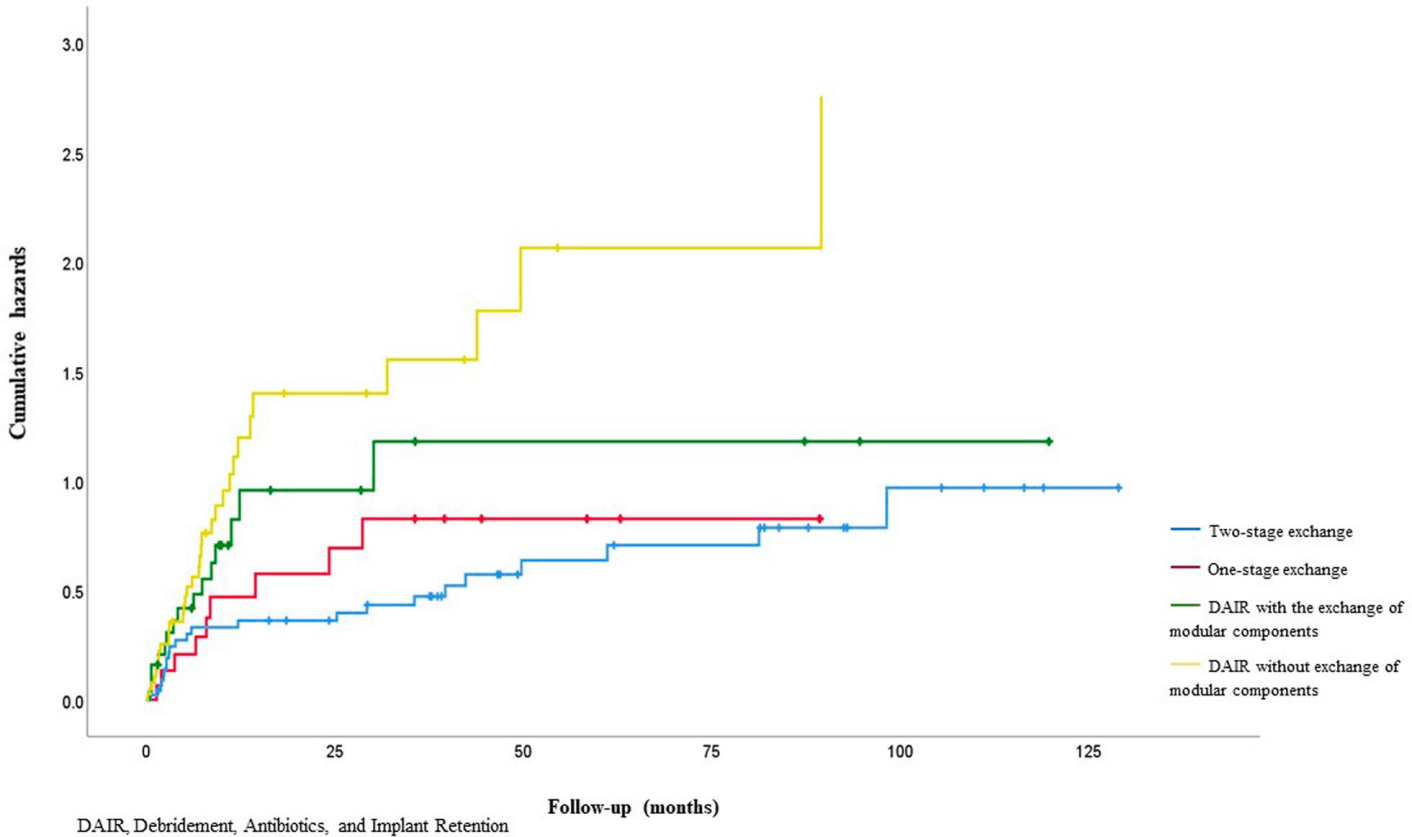
Kaplan–Meier curves showing the time to infection recurrence by type of surgical treatment

Following a one-stage procedure, success rates were 63.6% (95%CI 45.5–81.7) at 12 months, 45.5% (95%CI 36.4–58.8) at 2 years and 36.4% (95%CI 26.0–46.7) at 4 years (Fig. 3). In comparison, the corresponding values for two-stage exchanges, including those without reimplantation, were 71.2% (95%CI 61.8–80.6) at 12 months, 69.0% (95%CI 59.6–78.3) at 2 years and 55.3 (95%CI 46.9–63.8) at 4 years. The risk of recurrent infection was similar at 12 months for one and two-stage procedures. Success rates for completed two-stage exchange were higher at 82.8% (95%CI 71.4–94.1) at 12 months, 79.3% (95%CI 67.6–91.0) at 2 years and 61.0% (95%CI 49.1–72.9) at 4 years (Fig. 4).

The risk of recurrent infection following DAIRs with an exchange of modular components was 48.7% (95%CI 36.6–57.4) at 12 months, 44.6% (95%CI 36.6–52.7) at 2 years and 39.7% (95%CI 32.3–47.0) at 4 years. The corresponding values for DAIRs without exchange of modular components were 32.9% (95%CI 28.0–37.9) at 12 months, 24.7% (95%CI 21.3–28.1) at 2 years and 16.9% (95%CI 14.7–19.2) at 4 years.

There was no 30-day EPR infection-related mortality in the cohort.

### Risk factors for Recurrent Infection

**Fig. 5:**
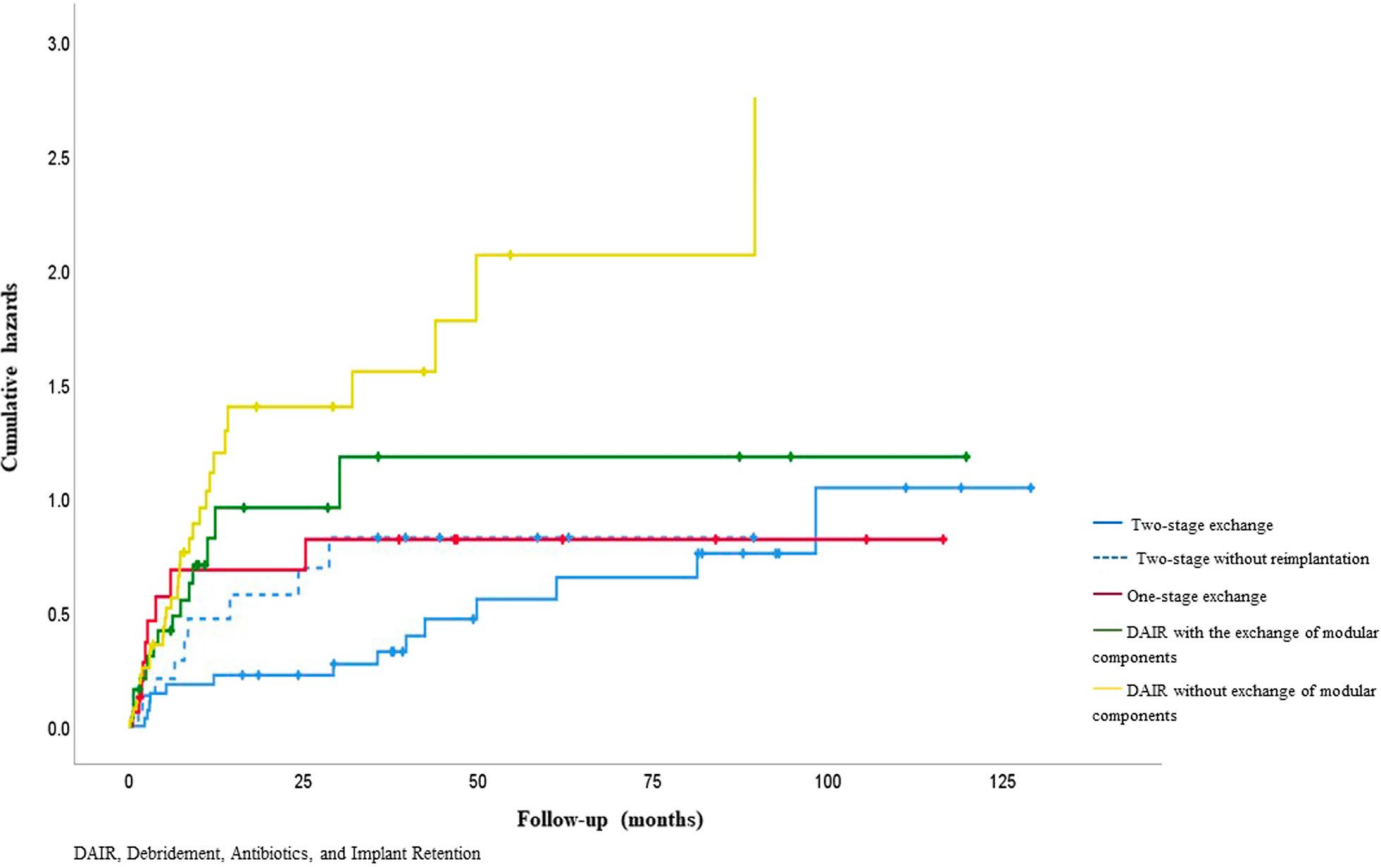
Kaplan–Meier curves showing the time to infection recurrence following surgical treatments by intention to treat

**Fig. 6:**
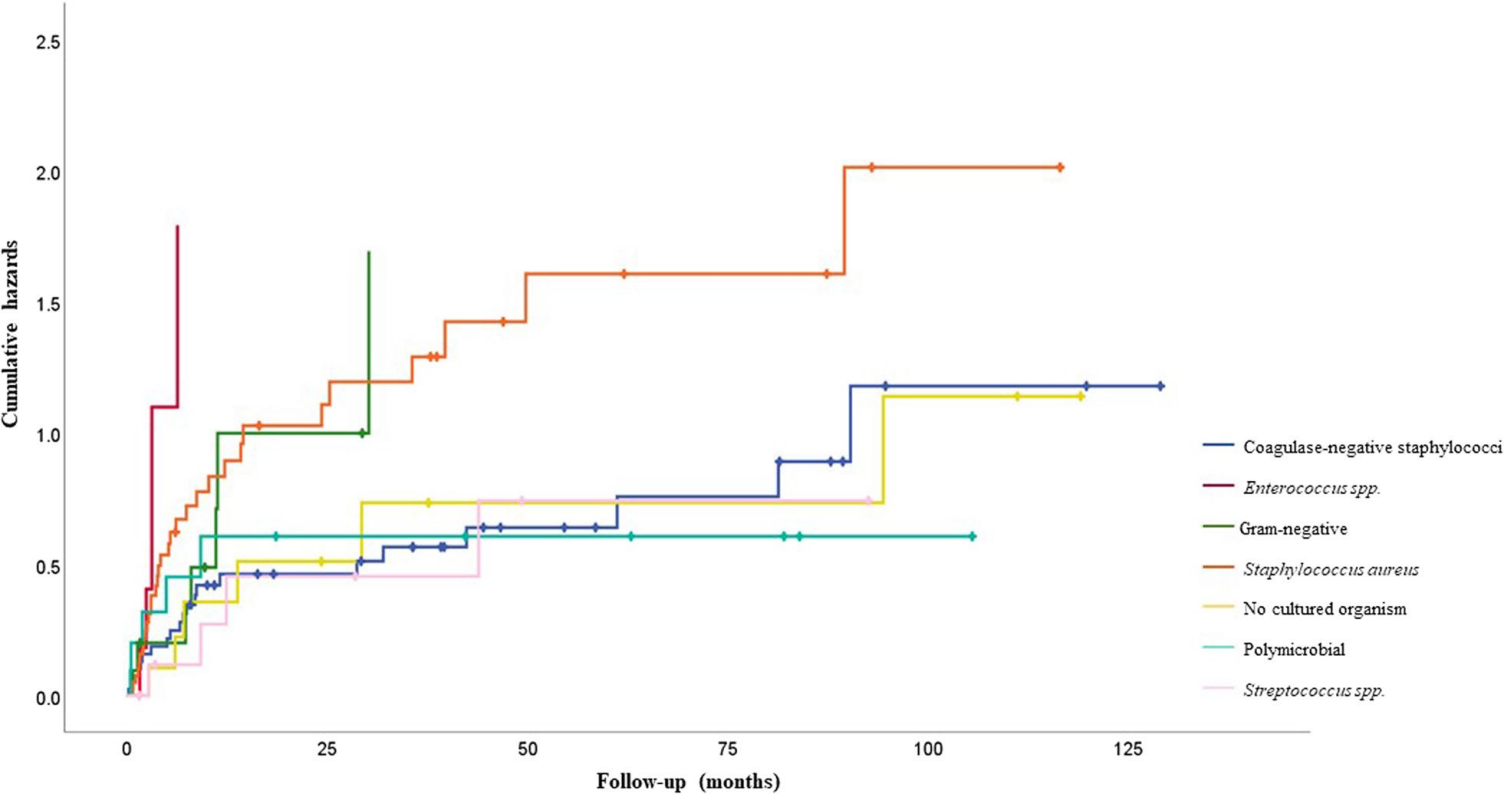
Kaplan–Meier curves showing time to infection recurrence by microorganism group

Significant risk factors for recurrent infection on univariate analysis were: type of surgical treatment, with DAIRs without exchange of modular components as the highest risk (HR 3.70: 95%CI 1.93–7.10); P = 0.002; infection due to *Enterococcus spp*. (HR 3.19: 95CI 1.51–6.74; *P* = 0.007) (Fig. 5); significantly compromised soft tissue status (HR 2.74: 95%CI 1.44–5.23; *P* = 0.008) (Fig. 6) and an increasing number of prior surgical treatments for infection (HR 2.64: 95%CI 1.39–5.01); *P* = 0.01). Reimplantation with a silver-coated prosthesis did not reduce the risk of further infection (HR 1.17: 95% CI 0.56–2.44; *P* = 0.68) (Table 1).

**Table 1:** Univariate analyses of factors associated with time to recurrent infection

Variable	Category/term	No. of infection recurrence/total	Hazard ratio (95% CI)	*P* value
Age ^(**)^	–	–	1.01 (1.00 – 1.02)	0.204
Gender	Female	30/48	1	0.348
	Male	53/82	1.24 (0.79 – 195)	
BMI ^(*)^	–	–	0.99 (0.95 – 1.02)	0.437
Charlson score	0	42/61	1	0.482
	1 – 2	26/46	0.74 (0.45 – 1.21)	
	3 +	15/23	0.88 (0.49 – 1.59)	
Tumour type	Osteosarcoma	46/65	1	0.434
	Chondrosarcoma	20/30	1.10 (0.65 – 1.86)	
	Ewing’s sarcoma	3/5	0.82 90.26 – 2.64)	
	Giant cell tumour of bone	5/9	0.59 (0.23- 1.49)	
	Metastatic bone disease	4/9	0.63 (0.23 – 1.75)	
	Spindle cell sarcoma	¾	1.28 (0.40 – 4.14)	
	Other sarcomas	2/8	0.34 (0.08 – 1.39)	
Type of prostheses	Distal femoral replacement	28/46	1	0.237
	Proximal tibial replacement	26/31	1.32 (0.81 – 2.18)	
	Proximal femoral replacement	10/21	0.65 (0.33 – 1.25)	
	Hemipelvic reconstruction	4/8	1.03 (0.40 – 2.64)	
	Total femoral replacement	6/7	1.11 (0.40 – 2.64)	
	Diaphyseal femoral replacement	2/6	0.42 (0.12 – 1.50)	
	Distal humeral replacement	¾	1.79 (0.61 – 5.19)	
	Proximal humeral replacement	3/5	2.14 (0.97 – 4.75)	
	Total radial replacement	1/2	0.85 (0.52 – 1.40)	
Prior revisions	0	52/90	1	**0.01**
	1	19/27	1.30 (0.77 – 2.19)	
	2 or more	12/13	2.64 (1.39 – 5.01)	
Local status	Uncompromised	16/33	1	**0.008**
	Compromised	43/69	1.58 (0.89 – 2.82)	
	Significantly compromised	24/28	2.74 (1.44 – 5.23)	
Radiotherapy	No	65/101	1	0.877
	Yes	18/29	1.04 (0.61–1.76)	
Chemotherapy within 3 months	No	72/107	1	0.283
	Yes	11/23	0.71 (0.37 – 1.33)	
Type of surgery	Two-stage exchange (complete)	13/29	1	**0.002**
	Two-stage exchange (without reimplantation)	9/17	1.74 (0.74 0 4.06)	
	One-stage exchange	7/11	1.87 (0.74 – 4.71)	
	DAIR with an exchange of modular components	20/33	2.23 (1.11 – 4.51)	
	DAIR without exchange of modular components	34/40	3.70 (1.93 – 7.10)	
	Excision arthroplasty ^(+)^	0/3		
Silver coated implant	No	12/25	1	0.684
	Yes	17/32	1.17 (0.56 – 2.44)	
Organisms	Coagulase-negative staphylococci	21/41	1	**0.007**
	*Staphylococcus aureus*	32/41	1.38 (0.92—2.05)	
	*Streptococcus spp.*	5/10	0.64 (0.31 – 1.34)	
	*Enterococcus spp.*	6/6	3.19 (1.51 – 6.74)	
	Gram-negatives	8/11	1.39 (0.73 – 2.67)	
	Polymicrobial	5/11	0.61 (0.28 – 1.35)	
	No cultured organism	6/10	0.66 (0.42 – 1.04)	

*Hazard ratio given for a 5-point increase in BMI, ** Hazard ratios given for a 10-year increase in age, ( +) Excluded from regression analysis as no reinfections in this category, (#) Unable to calculate as insufficient patients reached this point of infection recurrence, values in bold are significant (*p* < 0.1), DAIR debridement, antibiotics and implant retention

### Multivariate analysis

**Fig. 7:**
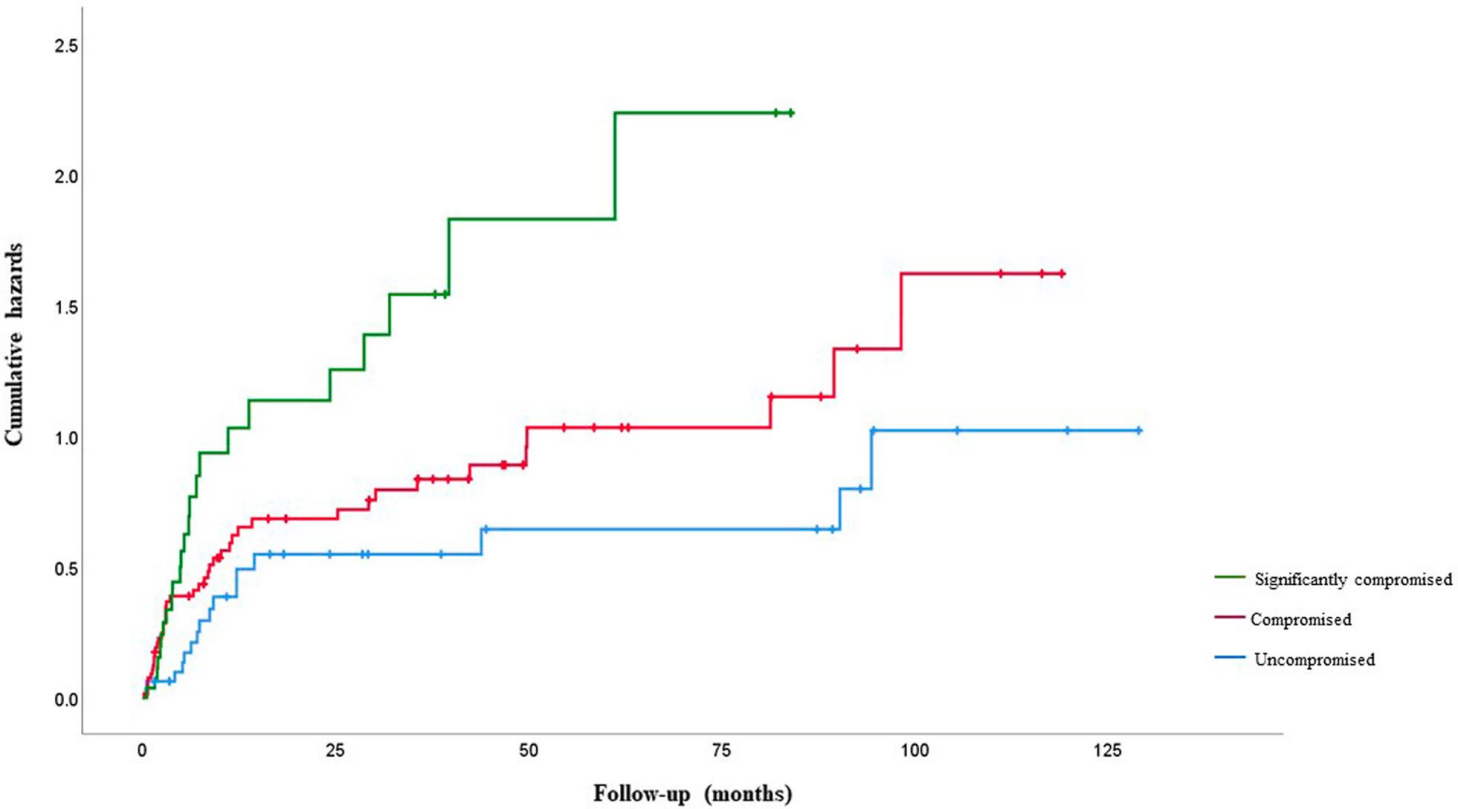
Kaplan–Meier curves showing time to infection recurrence by local soft tissue status graded according to McPherson criteria

Multivariate analysis showed the following independent risk factors for recurrent infection: type of surgical treatment (DAIR without exchange of modular components highest risk (HR 4.75: 95%CI 2.43–9.30; *P* < 0.001)); significantly compromised soft tissue status (HR 4.41: 95%CI 2.18–8.92; *P* < 0.001); infection involving *Enterococcus spp*. (HR 7.31: 95%CI 2.73–19.52); *P* = 0.001) (Table 2). The number of prior surgical treatments was not associated with further episodes of infection in the multivariate analysis (Fig. 7).

**Table 2:** Multivariable analyses of factors associated with time to recurrent infection

Variable	Category/term	Hazard ratio (95% CI)	*P* value
Type surgery	Two-stage exchange (with reimplantation)	1	** < 0.001**
	Two-stage exchange (no reimplantation)	1.53 (0.63 – 3.71)	
	One-stage exchange	2.57 (0.98 – 6.72)	
	DAIR with an exchange of modular components	4.14 (1.89 – 9.03)	
	DAIR without exchange of modular components	4.75 (2.43—9.30)	
	Excision arthroplasty ( +)	-	
Local status	Uncompromised	1	** < 0.001**
	Compromised	2.26 (1.22 – 4.19)	
	Significantly compromised	4.41 (2.18 – 8.92)	
Organisms	Coagulase-negative staphylococci	1	**0.001**
	*Staphylococcus aureus*	2.55 (1.43 – 4.56)	
	*Streptococcus spp.*	1.41 (0.52 – 3.52)	
	*Enterococcus spp.*	7.31 (2.73 – 19.52)	
	Gram negative	1.51 (0.65 – 3.49)	
	Polymicrobial	1.04 (0.38 – 2.87)	
	No cultured organism	1.34 (0.51 – 3.52)	

( +) Excluded from regression analysis as no reinfections in this category, values in bold are significant (*p* < 0.05), DAIR debridement antibiotics and implant retention

## Discussion

Our aim was to review the success of different approaches to the treatment of infected EPRs in our centre in order to improve outcomes and identify patients at high risk of recurrent infection. We have shown that compromised local soft tissue status, prior surgical treatments for infection, the type of surgery, and infection with *Enterococcus spp* are all associated with a higher risk of recurrent infection.

The association between poor soft tissues and recurrent infection has been described in several studies involving prosthetic joint infection (PJI) patients [4, 11]. Among our cohort, 75.1% of patients had at least one of the criteria associated with compromised soft tissues. Patients undergoing EPR for oncological conditions are more likely than others to have compromised soft tissues as the index procedure often requires resection of soft tissues including bone, and some patients have radiotherapy. The most common soft tissue problems in our cohort were sinus, radiotherapy damage, large dead space defects, and open wounds. The importance of soft tissue cover means there should be a low threshold for involving a plastic surgical team as part of multidisciplinary management.

The microorganisms associated with an infected EPR are similar to conventional PJI at first infection. These are predominantly coagulase-negative staphylococci (30 to 43%) and *Staphylococcus aureus* (12 to 23%), followed by polymicrobial infections (10 to 11%) and streptococci (9 to 10%). There are a small number of first infections with Gram-negative bacilli (3 to 6 per cent), enterococci (3 to 7%) and anaerobes (2 to 4%), but these become more common with subsequent episodes of infection, likely due to selection pressure after successive treatments with surgery and antibiotics. These microorganisms are often resistant to conventional antibiotics and are considered difficult to treat in PJI algorithms [12, 13]. Our data support the need for prosthesis removal where the infection is caused by *Enterococcus spp*.

We defined recurrent infection based on clinical findings regardless of whether the same pathogen was involved. 57% (24 of 42) of recurrent infections involved the same organisms as the preceding episode, suggesting that these represented relapses rather than new infections. The high overall relapse rate likely reflects the difficulty associated with two-stage exchange after EPR surgery, when the use of custom and very large implants makes the removal of an implant and the use of a spacer challenging.

Although silver-coated EPRs may have an antimicrobial effect, we did not find a reduction in the risk of recurrent infection, which is consistent with other studies [5].

When a second-stage reimplantation was performed, two-stage exchange had the highest success rates (82.8% at 12 months, 79.3% at 2 years and 61.0% at 4 years). Our results are comparable to others which report success rates of 63 to 100% [4–6]. Jeys et al. reviewed 58 patients treated with a two-stage exchange for infected EPR. 72% of patients were infection-free at 24 months [6]. Sigmund et al. reported a success rate of 62% at 48 months following a two-stage exchange [5]. The authors suggested their lower success rate was due to a lower proportion of patients (18 of 32 (56%)), having removal of all prosthetic stems. In their subgroup analysis, the success rate for patients who had removal of all implants was nearly three times higher than those who had a retained stem (22% vs 64%). In our series, all patients with a two-stage exchange had removal of all stems. 37% (17/46) of patients for whom a two-stage exchange was planned, did not have a second-stage reimplantation. The most common reasons for not undergoing a second-stage procedure were refusal by the patient, and concerns about severe medical comorbidities or poor soft tissues. When these patients are included, the success rate of patients selected for two-stage exchange is lower (71.2% at 12 months, 69.0% at 2 years and 55.3% at 4 years). Published studies which do not include this group may therefore overestimate the overall success rate of two-stage exchange. Nevertheless, our finding that a two-stage exchange is associated with the lowest risk of recurrent infection is consistent with the international consensus for the treatment of chronic EPR infection [14].

A one-stage exchange avoids the use of a spacer and is therefore attractive. Algorithms for revision arthroplasty recommend that one-stage exchange can be considered in early or low-grade infections with good soft tissue coverage and caused by pathogens sensitive to biofilm active antimicrobials [12, 13]. In revision knee arthroplasty, when these criteria are met, high success rates between 70 to 95% at 4 years have been reported [12]. Oncological EPR studies have lower success rates: Hardes et al. reported a 33% success rate at a mean follow-up of 32 months (0.3–128 months) and Jeys et al. 42% at a mean follow-up of 5.8 years (range 0.3–34 years) [6, 11]. Our success rates are broadly comparable, with 63.6% at one year, 45.5% at 2 years and 36.4% at 4 years. The high frequency of local soft tissue compromise and difficult-to-treat organisms, particularly in patients with a history of multiple revisions and oncological surgery, significantly limits the number of patients who meet the established criteria for one-stage exchange.

DAIR may be an attractive choice for well-fixed or custom, particularly joint-sparing implants. Success rates in PJI range from 50 to 70% in selected cases when specific criteria are met [12, 13]. These include early infections with symptom onset within 4 weeks, good soft tissue coverage and susceptible organism(s) sensitive to available antibiotics. The success rates in EPR studies are highly variable, between 39 and 70% [1, 15]. Most have small numbers and variable follow-up periods, and none provide a full description of the procedure. The success rate in our study was 44.6% at 2 years. As it may only be possible to change part of a large prostheses there may be limits to what can be achieved without exchanging all the implant components, particularly if the soft tissues are compromised.

DAIRs without exchange of modular components, when attempted as a definitive procedure for infection, were associated with the highest infection recurrence rate, of over 70% at 2 years. Although debridement may provide source control for sepsis, it is unlikely to eradicate infection and should not be considered a definitive treatment.

Our overall success rates are lower than published studies, which seldom include a large sample of salvage procedures and multiply treated EPRs [5, 6, 9]. We consider our study more representative of real-life clinical practice and provide important data that will better inform everyday decision-making. EPR infection remains difficult to manage and some patients inevitably require amputation. The overall amputation rate for infection of 21% in our study is similar to other studies [16].

Our findings have implications for the provision of services for patients with EPR infections. We recommend that patients be promptly assessed and investigated for a microbiological diagnosis and optimisation of soft tissues as part of an infection MDT before deciding on treatment intent.

Our study benefits from a relatively large cohort with long follow-up. All patients were included in the cohort, and none were lost to follow-up, minimising the risk of selection bias. Although a relatively large sample, this is a heterogeneous population undergoing a range of procedures for a wide range of conditions and our study is too small to detect small effects on outcome. Although the effect of confounding variables was considered, our study was retrospective which increases the possibility of unmeasured confounders.

## Conclusion

Infected EPR remains a significant challenge in orthopaedic oncology. Prior surgical treatment for infection, poor soft tissue status and infection with *Enterococcus spp*. are independent risk factors for recurrent infection after treatment. A two-stage exchange is the most successful procedure for eradicating infection. The overall success rate after one-stage exchange is limited by the high frequency of local soft tissue compromise. Debridement may control sepsis but is not a definitive treatment for infection. DAIR procedures vary significantly and are difficult to define in relation to massive EPRs. Identification of risk factors, timely assessment and a multidisciplinary approach should be available to manage these complex infections. Larger studies are needed to identify microbiological and surgical factors associated with the success of surgical treatment in orthopaedic oncology.

## Data Availability

The data sets used and/or analysed during the current study are available from the corresponding author upon reasonable request.

## References

[1] Dhanoa A, Ajit Singh V, Elbahri H (2015). Deep infections after endoprosthetic replacement operations in orthopedic oncology patients. Surg Infect.

[2] Henderson ER (2011). Failure mode classification for tumor endoprostheses: retrospective review of five institutions and a literature review. J Bone Joint Surg Ser A.

[3] Racano A, Pazionis T, Farrokhyar F, Deheshi B, Ghert M (2013). High infection rate outcomes in long-bone tumor surgery with endoprosthetic reconstruction in adults: a systematic review. Clinic Orthopaedics Related Res.

[4] Grimer RJ, Belthur M, Chandrasekar C, Carter SR, Tillman R (2002). Two-stage revision for infected endoprostheses used in tumor surgery. Clin Orthop Relat Res.

[5] Sigmund IK (2018). Efficacy of different revision procedures for infected megaprostheses in musculoskeletal tumour surgery of the lower limb. PLoS ONE.

[6] Jeys LM, Grimer RJ, Carter SR, Tillman RM (2005). Periprosthetic infection in patients treated for an orthopaedic oncological condition. JBJS.

[7] McPherson EJ (2002). Periprosthetic total hip infection: outcomes using a staging system. Clin Orthop Relat Res.

[8] Li H-K (2019). Oral versus intravenous antibiotics for bone and joint infection. N Engl J Med.

[9] Flint MN, Griffin AM, Bell RS, Wunder JS, Ferguson PC (2007). Two-stage revision of infected uncemented lower extremity tumor endoprostheses. J Arthroplasty.

[10] Funovics PT (2011). Management of septic complications following modular endoprosthetic reconstruction of the proximal femur. Int Orthop.

[11] Hardes J (2006). Characteristics and outcome of infections associated with tumor endoprostheses. Arch Orthop Trauma Surg.

[12] Osmon DR (2013). Diagnosis and management of prosthetic joint infection: clinical practice guidelines by the infectious diseases society of America. Clinic Infectious Diseases.

[13] Parvizi J (2018). The 2018 definition of periprosthetic hip and Knee infection: an evidence-based and validated criteria. J Arthroplasty.

[14] Strony J (2019). Musculoskeletal infection in orthopaedic oncology: assessment of the 2018 international consensus meeting on musculoskeletal infection. J Bone Joint Surg Am.

[15] Peel T (2014). Infective complications following tumour endoprosthesis surgery for bone and soft tissue tumours. Eur J Surg Oncol.

[16] Mavrogenis AF (2015). Infected prostheses after lower-extremity bone tumor resection: clinical outcomes of 100 patients. Surg Infect (Larchmt).

